# Crude Cell-Free Extract From *Deinococcus radiodurans* Exhibit Anticancer Activity by Inducing Apoptosis in Triple-Negative Breast Cancer Cells

**DOI:** 10.3389/fcell.2020.00707

**Published:** 2020-07-30

**Authors:** Illiyas Maqbool, M. Sudharsan, G. Kanimozhi, Sara T. Alrashood, Haseeb A. Khan, Nagarajan Rajendra Prasad

**Affiliations:** ^1^Department of Biochemistry and Biotechnology, Annamalai University, Chidambaram, India; ^2^Dharmapuram Gnanambigai Government Arts College for Women, Mayiladuthurai, India; ^3^Department of Pharmaceutical Chemistry, College of Pharmacy, King Saud University, Riyadh, Saudi Arabia; ^4^Department of Biochemistry, College of Sciences, King Saud University, Riyadh, Saudi Arabia

**Keywords:** *Deinococcus radiodurans*, triple-negative breast carcinoma, anticancer agents, apoptosis, bioactive compounds, GC-MS

## Abstract

Extremophilic organisms have the potential to tolerate extremely challenging environments of nature. This property can be accredited to its production of novel secondary metabolites that possess anticancer and other pharmaceutical values. The present study was aimed to investigate the anticancer activity of crude secondary metabolite extract (CSME) obtained from the radiation-tolerant bacterium *Deinococcus radiodurans* in triple-negative human breast carcinoma (MDA-MB-231) cells. The 3-(4, 5-dimethylthiazol-2-yl)-2, 5-diphenyltetrazolium bromide (MTT) assay showed the antiproliferative potential of CSME in MDA-MB-231 cells (IC_50_ = 25 μg/ml) and MCF-7 cells (IC_50_ = 10 μg/ml). Further, the CSME treatment led to the production of intracellular reactive oxygen species (ROS) and nuclear membrane alterations with the formation of apoptotic bodies in MDA-MB-231 cells. Considerable DNA damage and low antioxidant status were observed in CSME-treated MDA-MB-231 cells. The results also showed that the CSME treatment induced apoptotic markers expression in MDA-MB-231 cells. Western blot results illustrated significant upregulation of p53, caspase-3, and caspase-9 proteins expression. Then, we analyzed the presence of secondary metabolites which may be linked with antiproliferative potential of CSME by gas chromatography-mass spectrometry (GC-MS). The results illustrated the presence of 23 bioactive compounds some of which are already reported to possess anticancer properties. The study indicates that the CSME of *D. radiodurans* possess anticancer properties and exhibit the potential to be used as an anticancer agent.

## Introduction

Extremophiles are organisms that can survive extreme environmental conditions, such as those in deserts, hot vents, radiation-exposed environments, volcanic areas, extreme sea depths, and low oxygen environments (Kumar et al., 2010). The extremolytes, effective natural bioactive compounds, produced by these extremophiles maximize the efficacy and minimize the toxicity in cancer treatment (Azamjon et al., 2011; Coker, 2016). The production of unique pigments and secondary metabolites could offer them the capability to thrive in radiation-rich environments. These types of microbial secondary metabolites are an important source for discovering novel chemotherapeutic agents (Wohlleben et al., 2016; Raimundo et al., 2018). The bioactive compounds from extremophiles are believed to provide sources of therapeutical agents, especially antibiotics and effective anticancer drugs (Kumar et al., 2010). Among these extremophiles, *Deinococcus radiodurans* is an important organism because it can able to survive in high radiation, ionizing and non-ionizing exposure (Slade and Radman, 2011). *D. radiodurans* is a gram-positive and red-pigmented bacterium extremely resistant to several environmental conditions, such as gamma radiation, UV radiation, and oxidative stress (Krisko and Radman, 2013). Deinococcal exopolysaccharide (DeinoPol), a component of *D. radiodurans* cell wall, has been already reported to exhibit antioxidant properties. DeinoPol exerts highly protective effects on human keratinocytes in response to stress-induced apoptosis by effectively scavenging ROS (Lin et al., 2020). Thus, extremophiles appear to be good potential candidates for novel secondary metabolites.

Cancer is a leading cause of mortality, resulting in a large economic burden on the world population (Neelam et al., 2019). Breast cancer is one of the most common cancers in the world including countries such as India (Saranath and Khanna, 2014); 90% of cancer patients die due to the metastasis of cancer (Seyfried, 2012). The triple-negative breast cancer (TNBC) exhibit negative expression for estrogen receptor (ER), progesterone receptor (PR) as well as human epidermal growth factor receptor 2 (HER-2). This type of breast cancer is more prevalent in young women (12–17%) (Ouyang et al., 2014). The approaches to treat TNBC are limited and a major challenge for breast cancer drug discovery facing the breast cancer field (Reis-Filho and Tutt, 2008), making it necessary to formulate novel anticancer drugs. The survival strategies of extremophilic organisms are frequently accompanied by modifications of gene regulation and pathways of metabolic reactions, increasing the possibility of discovering pharmaceutically important novel and efficient metabolites (Park and Park, 2018). The *D. radiodurans* employs pyrroloquinoline quinone (PQQ) (He et al., 2003; Ishii et al., 2010) and carotenoids (deinoxanthine) as predominant secondary antioxidant metabolites (Khairnar et al., 2003). The crude secondary metabolites extract (CSME) obtained from some strains of marine bacteria has previously been reported to induce cytotoxic effects (ID_50_ = 7.20–19.84 μg/ml) and apoptosis in HeLa cells (Lin et al., 2005). The CSME (ethyl acetate extract) of some extremophilic bacteria has been reported to exhibit excellent anticancer properties which could be helpful to treat cancer (Haque et al., 2016). A study reported that n-butanol of CSMEs from microbial origin exhibited selective cytotoxicity and apoptosis against breast cancer (MCF-7) cells (Obeidat, 2017). The silver nanoparticles biosynthesized using *D. radiodurans* have also been reported to exhibit excellent anticancer activity against MCF-7 cell line (IC_50_ = 7–8 μg/ml) (Kulkarni et al., 2015). As the CSME of *D. radiodurans* possesses diverse secondary metabolites, it can be a useful extract for evaluating the chemotherapeutic activity. Gas chromatography-mass spectrometry (GC-MS) is a versatile technique used to identify individual components from complex mixtures (Stashenko and Martínez, 2012). The present study identifies the bioactive compounds in the CSME of *D. radiodurans* and investigates the anticancer activity of CSME against MDA-MB-231 cells.

## Materials and Methods

### Media and Reagents

Bacterial culture media was purchased from HiMedia, Mumbai. All cell culture media components, Dulbecco’s Modified Eagle’s Medium (DMEM), Fetal Bovine Serum (FBS), and antibiotics were purchased from HiMedia Laboratories, Mumbai. The cell culture assay reagents, trypsin, MTT, ethidium bromide, rhodamine-123, acridine orange, PBS, low-melting point agarose, EDTA, tris buffer, ammonium persulfate, and ethyl acetate, were purchased from Sigma Aldrich, India. The TUNEL APO-BrdU apoptosis kit was procured from Invitrogen.

### Study Plan

**FIGURE 1:**
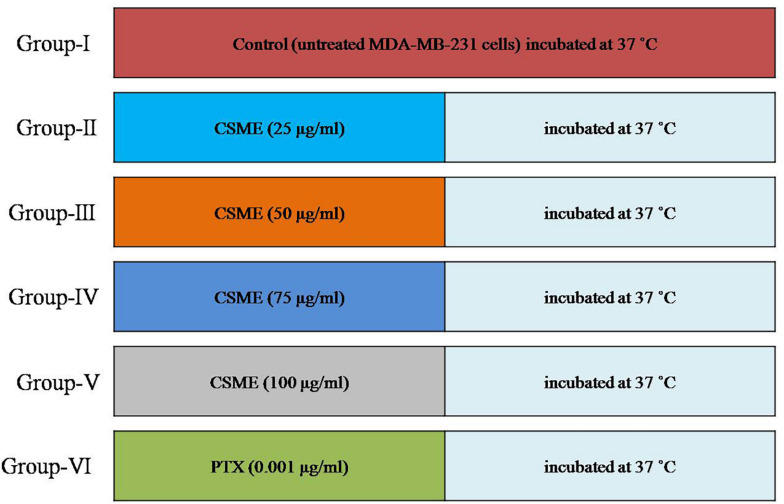
Study plan.

The treatment plan of CSME was followed according to the given order. After allowing MDA-MB-231 cells to grow for 24 h, the cells were divided into six experimental groups, i.e., (I) Untreated control cells, (II) Cells treated with 25 μg/ml CSME, (III) Cells treated with 50 μg/ml CSME, (IV) Cells treated with 75 μg/ml CSME, (V) Cells treated with 100 μg/ml CSME, and (VI) Cells treated with 0.001 μg/ml paclitaxel (PTX) (Figure 1). This treatment plan was carried out for each assay employed in this study unless otherwise mentioned.

### Bacterial Strains and Growth Conditions

*Deinococcus radiodurans* MTCC 4465 (Microbial Type Culture Collection, Chandigarh, India) culture was maintained on TGY (0.5% tryptone, 0.3% yeast extract, and 0.1% glucose) agar (supplemented with 1.5% agar). For secondary metabolite production, *D. radiodurans* cultures were grown in TGY broth. The flasks containing *D. radiodurans* cultures were incubated at 30°C in a shaker incubator at 220 rpm for 7 days for the production of secondary metabolites.

### Production, Extraction, and Preparation of CSME From *D. radiodurans*

After 7 days of incubation, the *D. radiodurans* cultures were collected in sterile polypropylene tubes and centrifuged at 5,000 rpm for 10 min. The supernatants containing the secondary metabolites were collected in fresh sterile polypropylene tubes. The secondary metabolites were extracted with ethyl acetate and subjected to the rotatory evaporator to eliminate the ethyl acetate solvent followed by lyophilization. Finally, the CSMEs obtained were dissolved in DMSO and further used for the evaluation of the anticancer activity.

### Cell Lines Maintenance and Growth Condition

Triple-negative human breast carcinoma (MDA-MB-231) cells and breast carcinoma (MCF-7) cells were procured from National Centre for Cell Science, Pune, India. The cells were maintained in DMEM medium supplemented with 10% fetal bovine serum, 50 units/ml penicillin–streptomycin at 37°C in a 5% CO_2_ incubator. Human peripheral blood lymphocytes (HPBLs) were used as normal cells to evaluate any potential CSME-induced toxicity in normal cells. HPBLs were grown in RPMI-1640 medium at 37°C in a 5% CO_2_ incubator.

### Determination of Cell Viability

The 3-(4,5-dimethylthiazil-2-yl)-2,5-diphenyltetrazolium bromide (MTT) assay was performed to evaluate the cytotoxicity of CSME in MDA-MB-231 cells and MCF-7 breast cancer cells (Khan et al., 2007). Cells (4 × 10^4^ cells/well) were seeded in 96-well plates and incubated at 37°C for 24 h. After 24 h of incubation, the cells were treated with different concentrations (0–100 μg/ml) of CSME and incubated at 37°C for 24 h. After treatment, the fresh medium containing MTT (5 mg/ml) was added to each well, and the plate was incubated for additional 4 h. The MTT was then removed very carefully, and cells were washed twice with PBS. The formazan crystals were dissolved by adding 100 μl of DMSO to each well. The absorbance was measured at 570 nm (Tecan, Austria). Untreated MDA-MB-231cells and MCF-7 cells were used as controls while human peripheral blood lymphocytes (HPBLs) were used as normal cells. Paclitaxel (PTX) (0–100 μg/ml) was used as a standard drug.

### Measurement of Intracellular ROS

The intracellular ROS levels in MDA-MB-231 cells were measured by DCFH-DA method (Spagnuolo et al., 2006). Briefly, MDA-MB-231 cells (5 × 10^6^ cells/well) were treated with or without different concentrations (25, 50, 75, and 100 μg/ml) of CSME for 24 h. A fresh DCFH-DA (1 μg/ml) solution was added to each well in the dark for another 30 min before observed for fluorescence intensity with the multimode reader (Excitation 480 nm and Emission 530 nm) (Tecan, Austria).

### Apoptotic Morphological Changes by Acridine Orange/Ethidium Bromide (AO/EtBr) Dual Staining

Acridine orange/ethidium bromide (AO/EtBr) double staining was performed to assess apoptotic changes (Baskić et al., 2006). Briefly, control and CSME-treated MDA-MB-231 cells were stained with 4 μg/ml acridine orange and 4 μg/ml ethidium bromide for 15 min. The stained cells were examined under the fluorescence microscope in both red and green channels (Floid Cell Imaging System, Invitrogen).

### Mitochondrial Membrane Potential (ΛΨ*m*) Assay

Mitochondrial membrane potential (ΛΨ*m*) in MDA-MB-231 cells was measured by rhodamine-123 dye (Emaus et al., 1986). MDA-MB-231 cells (5 × 10^6^ cells) were treated with different concentrations (25, 50, 75, and 100 μg/ml) of CSME for 24 h. Rhodamine-123 dye (1 μg/ml) was added 2 h before the end of the experiment. The cells were washed with PBS and analyzed under a fluorescence microscope (Floid cell imaging system, Invitrogen).

### Analysis of Nuclear Alterations by DAPI Staining

Nuclear alterations and apoptotic body formation are the hallmarks of cell death (Sandra et al., 2003). The MDA-MB-231 cells (5 × 10^6^ cells) were seeded in a 96-well plate and incubated for 24 h at 37°C. The cells were then treated with different concentrations (25, 50, 75, and 100 μg/ml) of CSME for 24 h. Apoptotic cells were determined by fluorescence microscopy analysis for morphological changes (Floid, Invitrogen). Nuclear alteration analysis of DAPI stained cells was done with fluorescence microscopy (Floid cell imaging system, Invitrogen).

### Measurement of Lipid Peroxidation in MDA-MB-231 Cells

Measurement of lipid peroxidation was assessed by thiobarbituric acid reactive substances (TBARS) (Esterbauer and Cheeseman, 1990). The MDA-MB-231 cells were treated with different concentrations (25, 50, 75, and 100 μg/ml) of CSME for 24 h. Cell suspension from each treatment group (0.1 ml) was mixed with 0.2 ml of 8.1% SDS and 3 ml of TBA reagent (equal ratios of 0.8% TBA and 20% acetic acid, pH 3.5). The final reaction volume was made up to 4 ml with distilled water and placed in a water bath at 95°C for 1 h. The chromogen extraction was performed with n-butanol and pyridine (15:1 v/v). The absorbance was measured at 530 nm by the multimode reader. TBARS were expressed in nM/mg proteins.

### Evaluation of Antioxidant Status in MDA-MB-231 Cells

The antioxidant status in MDA-MB-231 cells was assessed by colorimetric method. Cells were treated with different concentrations of CSME (25, 50, 75, and 100 μg/ml) for 24 h. The level of GSH was measured by the method of Ellman (1959). Superoxide dismutase (SOD) activity was measured according to the method of Kakkar et al. (1984), based on the inhibition of NADH-PMS-NBT complex formation. Catalase (CAT) activity was analyzed by measuring the H_2_O_2_ after incubating with a dichromate-acetic acid solution (Sinha, 1972). The glutathione peroxidases (GPx) activity was also assayed in the treated as well as control cells (Rotruck et al., 1973). Untreated cells were kept as control.

### Analysis of DNA Damage by Comet Assay

The alkaline comet assay was employed for the assessment of single strand DNA breaks in MDA-MB-231 cells (Singh et al., 1988). After different CSME concentration (25, 50, 75, and 100 μg/ml) treatment for 24 h, 5 μl of cell suspension (2 × 10^5^ cells) was embedded in an agarose matrix and the cells were lysed (2.5 M NaCl, 100 mM EDTANa_2_, 10 mM Tris, 1% sodium sarcosinate, 1% Triton X-100, 10% dimethyl sulfoxide, pH 10) overnight at 4°C. After lysis, the slides were placed into an alkaline solution (300 mM NaOH, 1 mM EDTA-Na2, pH 13) for 20 min at 4°C and subsequently electrophoresed for 20 min at 1 V/cm. Finally, the slides were neutralized in a 0.4-M Tris buffer (pH 7.5), stained with EtBr (10 μg/mL), and analyzed at 250 × magnification using an epifluorescence microscope (Nikon) connected to an image analysis system. The values of tail DNA and tail moment were used to measure the level of DNA damage.

### Terminal Deoxynucleotidyl Transferase-Mediated dUTP Nick-End Labeling Assay

After treatment with different CSME concentrations (25, 50, 75, and 100 μg/ml) for 24 h, cells were harvested and washed with PBS. DNA strand breaks in MDA-MB-231 cells were detected by the APO-BrdU TUNEL assay kit (Invitrogen) as per the manufacturer’s instructions. Samples were analyzed with fluorescent microscopy (Floid cell imaging system, Invitrogen).

### Protein Extraction and Western Blotting Analysis

The MDA-MB-231 cells after treatment with different concentrations (25, 50, 75, and 100 μg/ml) of CSME for 24 h were assessed by western blotting (Nakashima et al., 2013). The protein isolation was done by adding 0.6 ml of cold RIPA buffer [10 mM Tris (pH 7.5), 0.1% SDS, and 1% Triton × 100] and protease inhibitors cocktail (Sigma-Aldrich, Mumbai, India) to each treated as well as untreated cells. The cells were then scrap harvested and the cell lysate obtained was then centrifuged at 10,000 × g at 4°C for 10 min. The supernatants were collected and protein concentration was determined with Nanodrop spectrophotometer (Thermo Fisher Scientific). Each protein sample (50 μg) was added to the SDS sample buffer and denatured at 95°C for 5 min. Proteins were separated with 10% SDS-PAGE electrophoresis and transferred to a 0.45 μm nitrocellulose membrane. The membrane was blocked with 5% bovine serum albumin for 1 h and incubated with specific monoclonal antibodies at 4°C overnight. Then the membrane was incubated with secondary antibodies at room temperature for 1 h, washed with TBST three times and detected with a chemiluminescent detecting system (Bio-Rad).

### GC-MS Analysis of *D. radiodurans* Crude Extract

The identification of secondary metabolites in a crude extract of *D. radiodurans* was carried out by the GC-MS technique. The GC-MS analysis was performed in an Agilent 7890 system comprising an AOC-20i auto-sampler and a gas chromatograph interfaced to a mass spectrometer (GC-MS) equipped with an Elite-5MS (5% diphenyl/95% dimethyl polysiloxane) fused capillary column (30 × 0.25 μm ID × 0.25 μm df). For GC-MS detection, an electron ionization system was operated in electron impact mode with ionization energy of 70 eV. Helium gas (99.999%) was used as a carrier gas at a constant flow rate of 1 ml/min, and an injection volume of 2 μl was employed (a split ratio of 10:1). The injector temperature was maintained at 250°C, the ion-source temperature was 200°C, the oven temperature was programmed from 110°C (isothermal for 2 min), with an increase of 10°C/min to 200°C, then 5°C/min to 280°C, ending with a 9 min isothermal at 280°C. Mass spectra were taken at 70 eV; a scanning interval of 0.5 s and fragments from 45 to 450 Da. The solvent delay was 0 to 2 min, and the total GC-MS running time was 36 min. The relative percentage amount of each component was calculated by comparing its average peak area to the total areas. Turbo-Mass Gold-Perkin-Elmer-mass detector was used, and Turbo-Mass ver-5.2 software was used to handle mass spectra and chromatograms.

Interpretation of the GC-MS mass spectrum of the compounds identified was done using the database of National Institute Standard and Technology (NIST) having more than 62,000 patterns. The mass spectrum of the unknown component was compared with the spectrum of the known components stored in the NIST library. The name, molecular weight, and structure of the components of the test materials were confirmed.

### Statistical Analysis

The software SPSS (version 18.0) was used for statistical analysis. All error bars represent the standard error (SEM) of six independent experiments (*n* = 6) unless otherwise stated. Values not sharing a common superscript (a,b,c,d,e, and f) differ significantly at *P* < 0.05.

## Results

### Cytotoxicity of CSME in MDA-MB-231 and HPBL Cells

**FIGURE 2:**
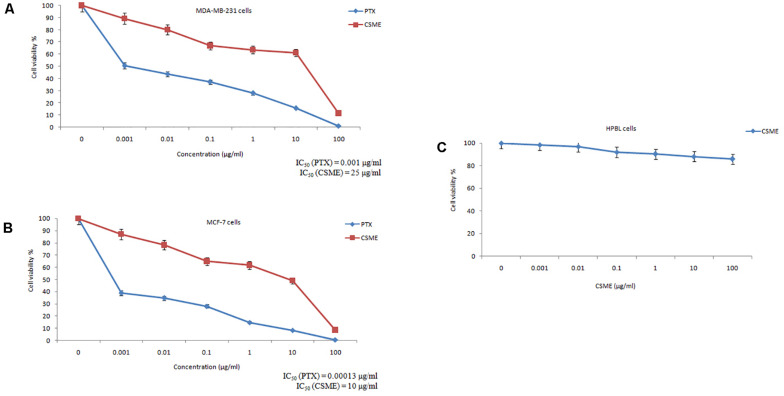
CSME exhibits cytotoxic effects in **(A)** MDA-MB-231 cells, **(B)** MCF-7 cells, and **(C)** HPBLs. Cells were treated with increasing concentrations of CSME ranging from 0.001 to 100 μg/ml for 24 h and then assessed for cell viability using MTT assay. Paclitaxel (0–100 μg/ml) was used as a standard anticancer drug in the assay. Values are given as mean ± SD of three experiments in each group. Values not sharing a common superscript (a,b,c,d,e, and f) differ significantly at *P* < 0.05 versus control (DMRT).

The present study investigated the cytotoxic potential of CSME obtained from *D. radiodurans* in MDA-MB-231 cells. A significant reduction in cell viability was found with different concentrations of CSME treatment in MDA-MB-231 cells (IC_50_ 25 μg/ml). Further, the cell viability in MDA-MB-231 cells was found to be decreased in a concentration-dependent manner when compared with untreated control MDA-MB-231 cells (Figure 2A). A similar reduction in cell viability was also found with different concentrations of CSME treatment in MCF-7 cells (IC_50_ 10 μg/ml). The results confirm that CSME exerts a significant cytotoxic effect in MDA-MB-231 and MCF-7 cells. However, our results illustrated that the CSME was more potent in MCF-7 cells than MD-MB-231 cell lines (Figure 2B). The treatment with PTX (standard anticancer drug) showed a much anticancer potential in both MDA-MB-231 (0.001 μg/ml) and MCF-7 (0.00015 μg/ml) cells.

Human peripheral blood lymphocytes (HPBLs) were employed as normal cells to determine any CSME-mediated cytotoxicity. No significant cytotoxicity was observed with different concentrations of CSME in HPBLs. We found a very little decrease in the% cell viability (85.7% viability) in HPBLs with 100 μg/ml of CSME treatment (Figure 2C). This showed that *D. radiodurans* CSME does not pose any harmful effects in normal cells.

### CSME Induces DNA Damage in MDA-MB-231 Cells

**FIGURE 3:**
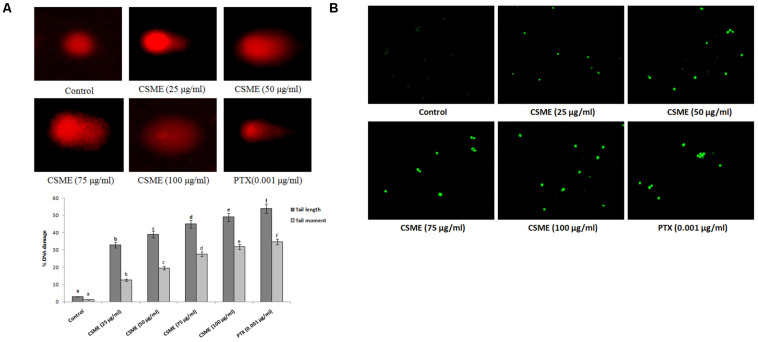
**(A)** CSME induces DNA damage in MDA-MB-231 cells. MDA-MB-231 cells were treated with different concentrations of CSME for 24 h. Cells lysates were electrophoresed and DNA strand breaks were detected by ethidium bromide staining using single-cell gel electrophoresis. The fluorescence micrographs show tail DNA and tail moment in CSME-treated MDA-MB-231cells. **(B)** TUNEL assay was carried out to detect CSME-induced DNA fragmentation in MDA-MB-231 cells. The images were taken under a fluorescence microscope (Floid cell imaging station, Invitrogen). Values are given as mean ± SD of six experiments in each group. Values not sharing a common marking (a,b,c,d,e, and f) differ significantly at *P* < 0.05 versus control (DMRT).

The present study employed single cell alkaline agarose gel-electrophoresis technique to know oxidative damages mediated single strand breaks (SSBs) caused by CSME treatment in MDA-MB-231 cells. We observed that the CSME treatment caused considerable DNA strand breaks in MDA-MB-231 cells. Further, we found that the DNA damage in MDA-MB-231 cells followed a concentration-dependent manner (Figure 3A). The percentage DNA damage was found to be 33%, 39%, 45%, and 49% for 25 μg/ml, 50 μg/ml, 75 μg/ml, and 100 μg/ml of CSME treatments, respectively. The treatment with 0.001 μg/ml of PTX was able to induce a DNA damage of 54% in MDA-MB-231 cells. Conversely, no significant DNA damage was observed in untreated control MDA-MB-231 cells (3%).

### CSME Treatment Induces Nuclear Fragmentation in MDA-MB-231 Cells

The effect of CSME from *D. radiodurans* on DNA fragmentation in MDA-MB-cells was carried out by terminal deoxynucleotidyl transferase dUTP nick end labeling (TUNEL) assay. In the present study, we observed a considerable level of nuclear fragmentation as a sign of apoptosis in MDA-MB-231 cells during different concentrations of CSME treatment when compared to untreated control cells. The CSME treatment induced a significant level of DNA fragmentation and nuclear bodies in MDA-MB-231 cells (Figure 3B). The DNA fragmentation was found to be increased with increase in the concentration of CSME treatment in MDA-MB-231 cells. All these results prove that CSME exhibits a considerable potential to induce apoptotic events in MDA-MB-231 cells.

### CSME Treatment Disturbs Antioxidant Potential in MDA-MB-231 Cells

**FIGURE 4:**
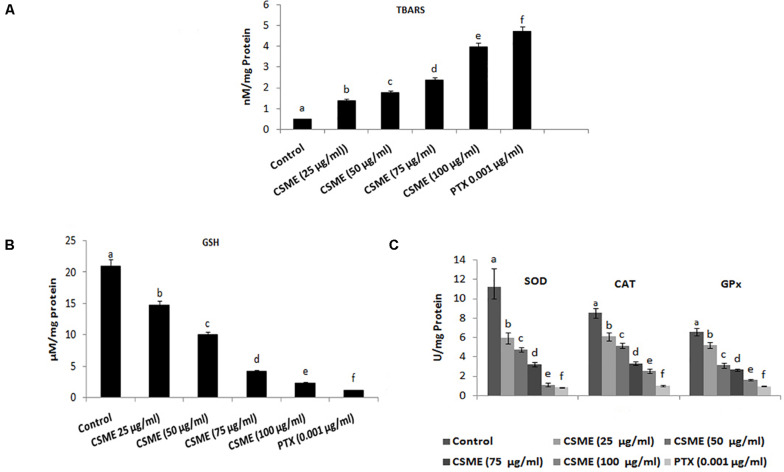
CSME treatment modulates lipid peroxidation and antioxidant status in MDA-MB-231 cells. MDA-MB-231 cells were treated with different concentrations of CSME for 24 h. Cells were collected and lipid peroxidation was assessed by measuring the levels of **(A)** TBARS and **(B)** GSH levels; **(C)** SOD, CAT, and GPx enzymatic activities. Values are given as mean ± SD of six experiments in each group. Values not sharing a common marking (a,b,c,d,e, and f) differ significantly at *P* < 0.05 versus control (DMRT).

The present study investigated the effect of CSME obtained from *D. radiodurans* on the lipid peroxidation and antioxidant status in MDA-MB-231 cells. The results show that the CSME treatment led to increased lipid peroxidation levels in MDA-MB-231 cells (Figure 4A). The level of lipid peroxidation was found to be 1.39, 1.78, 2.39 and 3.98 nM/mg protein for 25, 50, 75 and 100 μg/ml of CSME treatments, respectively. The treatment with 0.001 μg/ml of PTX was able to induce a lipid peroxidation of 0.48 nM/mg protein in MDA-MB-231 cells. No significant levels of lipid peroxidation were observed in untreated control MDA-MB-231 cells (0.48 nM/mg protein).

Moreover, the effect of CSME on the activities of several antioxidants such as reduced glutathione (GSH) as well as superoxide dismutase (SOD), catalase (CAT), and glutathione peroxidases (GPx) has also been studied. The levels of GSH were found to be 14.76, 9.97, 4.18, and 2.32 μM/mg protein with 25, 50, 75, and 100 μg/ml of CSME treatments, respectively in MDA-MB-231 cells. The treatment with 0.001 μg/ml of PTX significantly decreased the GSH activity (1.15 μM/mg protein) in MDA-MB-231 cells. No significant decrease in GSH activity was noticed in untreated control MDA-MB-231 cells (20.96 μM/mg protein) (Figure 4B).

Further, we noticed a significant reduction in SOD, CAT as well as GPx antioxidant activities during different concentrations of CSME treatment in MDA-MB-231 cells. The SOD activity was found to be 5.95, 4.75, 3.24, and 1.12 μg/ml for 25, 50, 75, and 100 μg/ml of CSME treatments, respectively in MDA-MB-231 cells. The treatment with 0.001 μg/ml PTX significantly decreased the SOD antioxidant enzyme activity to 0.84 μg/ml in MDA-MB-231 cells. No significant decrease in the SOD activity (11.18 μg/ml) was noticed in untreated control MDA-MB-231 cells. Also, the activity of CAT antioxidant enzyme activity was noticed to be reduced to 6.1, 5.14, 3.3, and 2.52 μg/ml for 25, 50, 75, and 100 μg/ml of CSME treatments, respectively, in MDA-MB-231 cells. The treatment with 0.001 μg/ml of PTX decreased the CAT antioxidant enzyme activity to 1.02 μg/ml in MDA-MB-231 cells. No significant reduction (8.53 μg/ml) in CAT antioxidant enzyme activity was noticed in untreated control MDA-MB-231 cells. Further, reduction in GPx antioxidant enzyme activity was noticed to be 5.18, 3.12, 2.66, and 1.61 μg/ml for 25, 50, 75, and 100 μg/ml of CSME treatments, respectively in MDA-MB-231 cells. The treatment with 0.001 μg/ml of PTX reduced the GPx antioxidant enzyme activity to 0.96 μg/ml in MDA-MB-231 cells. No significant reduction (6.57 μg/ml) in GPx antioxidant enzyme activity was noticed in untreated control MDA-MB-231 cells (Figure 4C). These results prove that *D. radiodurans* CSME disturbs the antioxidant status in MDA-MB-231 cells.

### CSME-Mediated Production of ROS and Alteration of Mitochondrion Membrane Potential in MDA-MB-231 Cells

**FIGURE 5:**
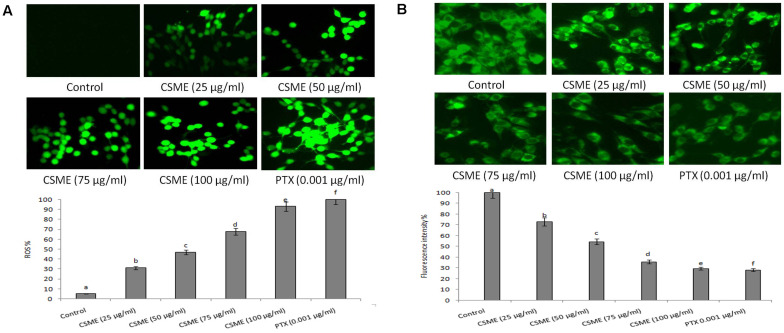
CSME treatment induces ROS and alterations in mitochondrial membrane potential in MDA-MB-231 cells. Photomicrograph shows **(A)** enhanced fluorescence intensity in MDA-MB-231 cells after treatment with different concentrations of CSME. **(B)** MDA-MB-231 cells after treatment with different concentrations of CSME displayed diminished green fluorescence. The cells were observed under a green lamp (Floid cell imaging system, Invitrogen). Bar diagram represents fluorescence intensity observed under excitation and emission at 485 and 530 nm, respectively, using a multimode reader (Tecan, Austria). Values are expressed as mean ± SD of six experiments in each group. Values not sharing a common marking (a,b,c,d,e, and f) differ significantly at *P* < 0.05 (DMRT).

The CSME obtained from *D. radiodurans* was investigated for its potential to induce intracellular ROS in MDA-MB-231 cells. The results from fluorescence microscopic examination clearly showed formation of intracellular ROS in CSME treated MDA-MB-231 cells (Figure 5A). Our results show that the CSME induced intracellular ROS in a concentration-dependent manner in MDA-MB-231 cells. The% ROS was found to be 31.21%, 47%, 67.98%, and 93% with 25, 50, 75, and 100 μg/ml of CSME treatments, respectively, in MDA-MB-231 cells. The treatment with 0.001 μg/ml of PTX was able to induce 100% ROS in MDA-MB-231 cells. No significant ROS levels were observed in untreated MDA-MB-231 cells (5.16%).

Further, we also observed a considerable loss of mitochondrial membrane potential (ΛΨ*m*) after CSME treatment in MDA-MB-231 cells (Figure 5B). The loss of ΛΨ*m* followed a concentration-dependent manner. The loss of ΛΨ*m* was observed as a decrease in the fluorescence intensity with increase in the CSME treatment concentration. The decrease in the ΛΨ*m* was found to be 72.9%, 54.41%, 35.7%, and 29.54% with 25, 50, 75, and 100 μg/ml of CSME treatments, respectively, in MDA-MB-231 cells. The treatment with 0.001 μg/ml of PTX was able to induce 28.21% loss of ΛΨ*m* in MDA-MB-231 cells. However, no significant loss of ΛΨ*m* (100%) was observed in untreated control MDA-MB-231 cells. The results suggest that CSME induces antioxidant instability which leads to the loss of ΛΨ*m* in MDA-MB-231 cells.

### CSME Treatment Induces Apoptotic Morphological Changes in MDA-MB-231 Cells

**FIGURE 6:**
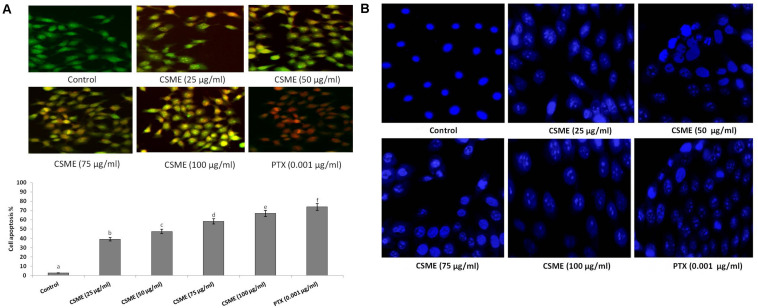
CSME induces apoptotic morphological changes and nuclear condensation in MDA-MB-231 cells. **(A)** CSME-treated cells displayed typical morphological changes and **(B)** fragmented nucleus. Images were taken under a fluorescence microscope (Floid cell imaging station, Invitrogen). Bar diagram represents% apoptotic cell death in different experimental groups. Values are given as mean ± SD of six experiments in each group. Values not sharing a common marking (a,b,c,d,e, and f) differ significantly at *P* < 0.05 (DMRT).

The CSME obtained from *D. radiodurans* was assessed for its potential to induce apoptotic morphological alterations in MDA-MB-231 cells. We observed that the CSME treatment was able to induce initial events of apoptosis (yellowish-orange chromatin) with some cells undergoing cell death (red chromatin) (Figure 6A). The CSME treatments induced significant apoptotic morphological alterations in MDA-MB-231 cells. The apoptotic effects of CSME in MDA-MB-231 cells were noticed to follow a concentration-dependent manner. The percentage apoptosis were found to be 38.92%, 47.41%, 58.22%, and 66.85% with 25, 50, 75, and 100 μg/ml of CSME treatments, respectively, in MDA-MB-231 cells. The treatment with 0.001 μg/ml of PTX was able to induce an apoptosis of 73.85% in MDA-MB-231 cells. However, no significant level of apoptosis (2.71%) was observed in untreated control MDA-MB-231 cells.

Furthermore, we investigated the effect of *D. radiodurans* CSME treatment on nuclear alterations in MDA-MB-231 cells by DAPI staining. We observed that the CSME from *D. radiodurans* induced considerable changes in nuclear and chromatin morphologies in MDA-MB-231 cells. The level of change in nuclear and chromatin morphologies with CSME treatment followed a concentration dependent manner. A higher level of these changes was observed with 100 μg/ml of CSME treatment in MDA-MB-231 cells. Conversely, no such changes were observed in untreated control MDA-MB-231 cells (Figure 6B).

### CSME Treatment Mediates Apoptotic Markers Expressions in MDA-MB-231 Cells

**FIGURE 7:**
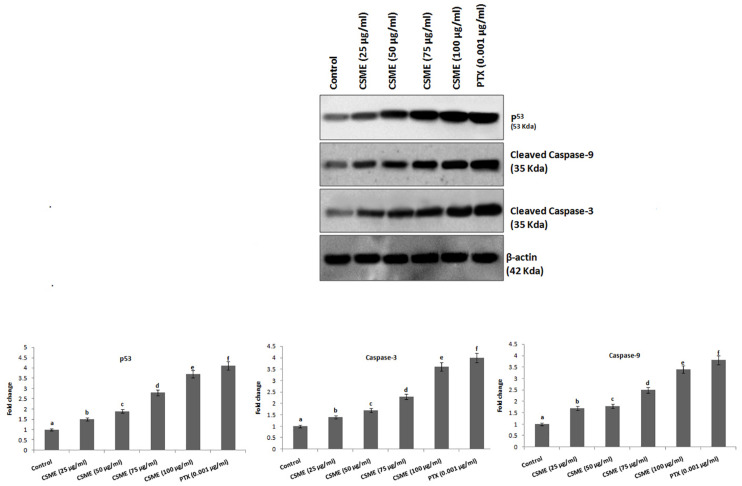
CSME induces upregulation of apoptotic markers expression in MDA-MB-231 cells. Western blot analysis was carried out to demonstrate the apoptotic effect of CSME treatment in MDA-MB-231 cells. Immunoblots were performed with cell lysate to determine the apoptotic protein expression such as p53, cleaved caspase-9, and cleaved caspase-3 by western blot analysis. Protein quantification was performed by densitometry analysis in Image Studio Software (LI-COR). Expressions were normalized with β-actin as the mean ± SD. *P* < 0.05 versus untreated control MDA-MB-231 cells from three independent experiments.

The effect of CSME treatment was studied for its potential to induce apoptotic marker expressions in MDA-MB-231 cells. The results showed that CSME treatment induced upregulation of p53, cleaved caspase-3 and cleaved caspase-9 protein expressions in MDA-MB-231 cells. We found a fold change of 1.5, 1.9, 2.8, and 3.7 with 25, 50, 75, and 100 μg/ml of CSME treatments, respectively in mDA-MB-231 cells. The treatment with 0.001 μg/ml of PTX induced a fold change of 4.1 in MDA-MB-231 cells. Further, we found a fold change of 1.7, 1.8, 2.5, and 3.4 with 25, 50, 75, and 100 μg/ml of CSME treatments, respectively in MDA-MB-231 cells. The treatment with 0.001 μg/ml of PTX induced a fold change of 3.8 in MDA-MB-231 cells. Also, a fold change of 1.4, 1.7, 2.3, and 3.6 was noticed with 25, 50, 75, and 100 μg/ml of CSME treatments, respectively in MDA-MB-231 cells. The treatment with 0.001 μg/ml of PTX induced a fold change of 4 in MDA-MB-231 cells (Figure 7). Conversely, we did not observe any significant expressions of these markers in untreated control MDA-MB-231 cells. The data from this study prove that the CSME treatment notably induces apoptosis through upregulation of apoptotic markers expression in MDA-MB-231 cells.

### GC-MS Data of *D. radiodurans* Crude Extract

**FIGURE 8:**
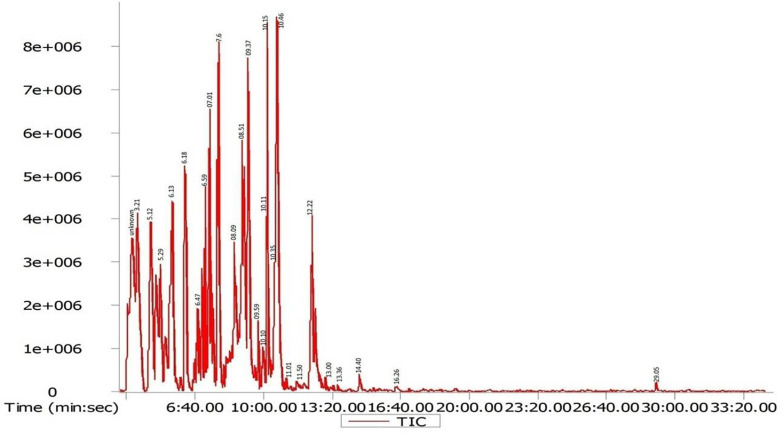
GC-MS chromatogram of different secondary metabolites identified in the crude secondary metabolite extract of *D. radiodurans*.

Gas chromatography-mass spectrometry chromatogram of the *D. radiodurans* crude extract showed 25 peaks indicating the presence of 25 compounds (Figure 8). On comparison of the mass spectra of each compound with the NIST library, the identification of bioactive constituents was carried out. The molecular formula, exact mass, retention time and peak area of all the identified compounds are listed (Table 1). Some of these compounds have already been identified as efficient anticancer compounds.

**TABLE 1:** Secondary metabolites identified in crude secondary metabolite extract from *D. radiodurans*.

Peak #	Name of the compound	Structure/formula	Exact mass	R.T (min)	Peak area %
1	Methoxyacetic acid, octyl ester	C_11_H_22_O_3_	202.1569	03:21.2	0.8463
2	3-O-Acetyl-exo-1,2-O-ethylidene-à-d-erythrofuranose	C_8_H_12_O_5_	188.0685	05:12.8	0.47445
3	11-Methyldodecanol	C_13_H_28_O	200.214	05:29.9	1.7422
4	Pentadecane, 2,6,10-trimethyl-	C_18_H_38_	254.2974	06:13.2	2.4736
5	Hexethal	C_12_H_20_N_2_O_3_	240.1474	06:18.8	0.009556
6	2,5-Cyclohexadiene-1,4-dione, 2,6-bis(1,1-dimethylethyl)-	C_14_H_20_O_2_	220.1463	06:47.7	0.36231
7	Eicosane	C_20_H_42_	282.3287	07:01.2	0.77193
8	Unknown 1	C_9_H_13_NO_2_	167.0946	07:06.9	0.028571
9	Heptadecane, 2,3-dimethyl-	C_19_H_40_	268.313	08:09.4	0.11541
10	Cyclodocosane, ethyl-	C_24_H_48_	336.3756	08:51.4	0.15977
11	Tetradecanoic acid, trimethylsilyl ester	C_17_H_36_O_2_Si	300.2485	09:37.9	0.077591
12	Methylamine, N, N-dimethyl-	C_3_H_9_N	59.0735	09:59.6	0.077363
13	Heptacosanoic acid, methyl ester	C_28_H_56_O_2_	424.428	10:10.5	1.6676
14	7,9-Di-tert-butyl-1-oxaspiro(4,5)deca-6,9-diene-2,8-dione	C_17_H_24_O_3_	276.1725	10:11.3	2.7288
15	Methoxyphenamine	C_11_H_17_NO	179.131	10:15.2	0.006498
16	L-Proline, N- valeryl-, hexyl ester	C_16_H_29_NO_3_	283.2147	10:35.2	1.2471
17	Hentriacontane	C_31_H_64_	436.5008	10:46.3	0.063137
18	D-Glucitol, 1-amino-1-deoxy-	C_6_H_15_NO_5_	181.095	11:01.8	0.004158
19	Pentacosanoic acid, 4- methyl-, methyl ester	C_27_H_54_O_2_	410.4124	11:50.9	0.018929
20	n-Hexadecanoic acid	C_16_H_32_O_2_	256.2402	12:22.7	3.4427
21	1-Dodecanol, 2-hexyl-	C_18_H_38_O	270.2923	13:00.1	0.1121
22	Heptadecane, 2,6,10,15-tetramethyl-	C_21_H_44_	296.3443	13:36.2	0.04829
23	9-Non-adecene	C_19_H_38_	266.2974	14:40.0	0.15637
24	Phthalic acid, 2-ethylhexyl isohexyl ester	C_22_H_34_O_4_	362.2457	16:26.7	0.030868
25	Unknown 27	C_44_H_62_N_4_	646.4974	29:05.7	0.032438

## Discussion

Among polyextremophiles, radiation-resistant organisms grow and survive at extreme exposures to ionizing as well as non-ionizing radiation (Rothschild and Mancinelli, 2001). *D. radiodurans* show very high resistance toward higher levels of gamma-radiation, UV-radiation, desiccation and oxidative stress (Krisko and Radman, 2013). In this study, we tested the cytotoxicity of CSME obtained from *D. radiodurans* in MDA-MB-231 cells and HPBL cells. The IC_50_ value of CSME in MDA-MB-231 cells was found to be 25 μg/ml while in MCF-7 cells, the IC_50_ value of CSME was found to be 10 μg/ml. Our results illustrate that MCF-7 cells were more sensitive than MDA-MB-231 cells. Previous results also illustrated those triple-negative MDA-MB-231 cells which are in general drug resistant (Li et al., 2012). No considerable decrease in cell viability was observed in normal HPBL cells with CSME treatment. Genome-wide transcriptome analyses illustrate *D. radiodurans* possess genetic make-up to produce several valuable secondary metabolites (Luan et al., 2014) which might be the reason to inhibit the proliferation of MDA-MB-231 cells. The antiproliferative activity of pigments produced by *D. radiodurans* has already been documented in Neuro-2a, Saos-2, HepG2, PC-3, and HT-29 cells (Choi et al., 2014; Tapia et al., 2019).

The formation of intracellular ROS is a hallmark of oxidative stress (Saraste and Pulkki, 2000). We found that CSME treatment-induced ROS generation in MDA-MB-231 cells. The results suggest that the secondary metabolites present in CSME of *D. radiodurans* induce ROS and therefore it leads to oxidative DNA damage in MDA-MB-231 cells. Pyrroloquinoline quinone (PQQ) is an antioxidant isolated from *D. radiodurans* (Lim et al., 2018; Illiyas et al., 2019). Quinones are highly active redox molecules that can form semiquinone radical anions in the acidic cancer cell environment which leads to the formation of ROS which ultimately damages cellular DNA (Bolton and Dunlap, 2016).

Antioxidant enzymes have a very important role in the oxidative stress defense in the cellular environment (Kurutas, 2015). Any disturbance in the antioxidants status and lipid metabolism has been known to be potentially deleterious for cellular function, having cytotoxic effects, damage to the cell membrane and cell apoptosis (Ayala et al., 2014). We observed increased TBARS levels in CSME treated MDA-MB-231 cells with a concomitant decrease of GSH, SOD and CAT enzyme activities. Our results demonstrate that CSME from *D. radiodurans* induces lipid peroxidation and depletion of antioxidant status in MDA-MB-231 cells. DNA damage has been reported to be a hallmark of cell senescence. DNA damage is a multi-factorial cellular phenomenon (Greenberg et al., 2006). It has also been reported that cellular DNA damage is also induced by the direct attack of DNA by increased levels of intracellular ROS generation (Nita and Grzybowski, 2016). The CSME treatment induced significant DNA single stand breaks in MDA-MB-231 cells. We also noticed that the CSME (25 μg/ml) treatment was enough to induce considerable DNA fragmentation in MDA-MB-231 cells. These results prove that CSME from *D. radiodurans* induced DNA damage which is due to oxidative stress in MDA MB-231 cells.

Apoptosis is believed to be the reason as well as the solution for tackling the problem of cancer as many new drugs are being employed which can target diverse aspects of apoptosis and finally cell death in cancer cells (Wong, 2011). The CSME from *D. radiodurans* induces apoptosis in MDA-MB-231 cells in a concentration-dependent manner. Nuclear fragmentation and condensation in cells is a characteristic of apoptotic induction (Toné et al., 2007). The loss of mitochondrial membrane potential (ΔΨm) is as an early event in the cell apoptosis process (Ly et al., 2003). The CSME treatment (25 μg/ml) was sufficient to induce significant nuclear fragmentation and condensation in MDA-MB-231 cells. The results established that CSME causes cell death by initiating the apoptosis process in MDA-MB-231 cells following a dose-dependent manner.

The levels of p53, cleaved caspase-3, and cleaved caspase-9 increased in MDA-MB-231 cells post CSME treatment. This suggests that the initiation of intracellular apoptotic events with CSME treatment is linked with the caspase-3 activation. Treating cancer cells with CSME significantly altered the expression of the mitochondrial death pathway. These results indicate that caspase-3-dependent apoptosis is associated with modulation of the Bcl-2/Bax protein ratio. In the present study, CSME obtained from *D. radiodurans* was investigated for its apoptotic activity. Earlier, Min et al. (2014) reported that pyrroloquinoline quinine, a major secondary metabolite of *D. radiodurans* induces cancer cell apoptosis via a mitochondrial-dependent pathway and down-regulating cellular bcl-2 protein expression. Similarly, Wen et al. (2018) reported that pyrroloquinoline quinine (PQQ) induces chondrosarcoma cell apoptosis by increasing intracellular reactive oxygen species. Wu et al. (2018) also showed that pyrroloquinoline quinone exhibits apoptotic properties in chondrosarcoma cells through activation of the mitochondrial caspase-dependent and caspase-independent pathways. The majority of bioactive compounds identified by GC-MS in the crude extract of *D. radiodurans* have previously been reported to exhibit anticancer properties. The ethyl cyclodocosane has been reported to possess antitumor and antiproliferative activity against HeLa cell line (Alkhatib et al., 2019). n-hexadecanoic acid that we found in the GC-MS analysis has also been earlier proved to show anticancer activity in HCT-116 cells (Ravi and Krishnan, 2017). 2,6,10,15-tetramethyl heptadecane was found to be involved in the metabolism of ovarian cancer stem cells (Vermeersch et al., 2014). Hentriacontane and methoxyacetic acid octyl ester present in *D. radiodurans* CSME have also been reported to exhibit antioxidant, anti-inflammatory and anti-tumor properties (Kim et al., 2011; Hailu et al., 2017). Therefore, this study proves that CSME of *D. radiodurans* could be a potential source of bioactive compounds that could be explored for anticancer properties at the molecular level.

## Conclusion

We demonstrated that CSME induced apoptosis in MDA-MB-231 cells through the upregulation of apoptotic markers expression. The results prove that the CSME of *D. radiodurans* can serve as a potential therapeutic agent against breast cancer. This is a novel finding since this type of activity has not been reported so far with the CSME of *D. radiodurans* strain employed in the present study. However, further studies are required to study the anticancer properties of CSME of *D. radiodurans* in other types of cancers.

## Data Availability Statement

The datasets presented in this article are not readily available because confidential. Requests to access the datasets should be directed to NP, drprasadnr@gmail.com.

## Author Contributions

IM and MS performed the experiments. SA and HK wrote the rough draft of the manuscript. NP and GK designed the concept and experiments. NP wrote the final version of the manuscript. All authors contributed to the article and approved the submitted version.

## Conflict of Interest

The authors declare that the research was conducted in the absence of any commercial or financial relationships that could be construed as a potential conflict of interest.
